# AI-Enabled Advanced Development for Assessing Low Circulating Blood Volume for Emergency Medical Care: Comparison of Compensatory Reserve Machine-Learning Algorithms

**DOI:** 10.3390/s22072642

**Published:** 2022-03-30

**Authors:** Victor A. Convertino, Robert W. Techentin, Ruth J. Poole, Ashley C. Dacy, Ashli N. Carlson, Sylvain Cardin, Clifton R. Haider, David R. Holmes III, Chad C. Wiggins, Michael J. Joyner, Timothy B. Curry, Omer T. Inan

**Affiliations:** 1 Battlefield Health & Trauma Center for Human Integrative Physiology, US Army Institute of Surgical Research, JBSA Fort Sam Houston, San Antonio, TX 78234, USA; ashli.n.carlson.mil@mail.mil; 2 Department of Medicine, Uniformed Services University of the Health Sciences, Bethesda, MD 20814, USA; 3 Department of Emergency Medicine, University of Texas Health, San Antonio, TX 77030, USA; 4 Special Purpose Processor Development Group, Mayo Clinic, Rochester, MN 55902, USA; techentin.robert@mayo.edu (R.W.T.); poole.ruth@mayo.edu (R.J.P.); haider.clifton@mayo.edu (C.R.H.); 5 Naval Medical Research Unit-San Antonio, JBSA Fort Sam Houston, San Antonio, TX 78234, USA; ashley.c.dacy.civ@mail.mil (A.C.D.); sylvain.cardin.civ@mail.mil (S.C.); 6 Biomedical Analytics and Computational Engineering Laboratory, Mayo Clinic, Rochester, MN 55902, USA; holmes.david3@mayo.edu; 7 Department of Anesthesiology and Perioperative Medicine, Mayo Clinic, Rochester, MN 55902, USA; wiggins.chad@mayo.edu (C.C.W.); joyner.michael@mayo.edu (M.J.J.); curry.timothy@mayo.edu (T.B.C.); 8 School of Electrical and Computer Engineering, Georgia Institute of Technology, Atlanta, GA 30332, USA; inan@gatech.edu

**Keywords:** hemorrhage, shock, medical monitoring, compensatory reserve, machine learning, deep learning, artificial intelligence, sensor signals

## Abstract

The application of artificial intelligence (AI) has provided new capabilities to develop advanced medical monitoring sensors for detection of clinical conditions of low circulating blood volume such as hemorrhage. The purpose of this study was to compare for the first time the discriminative ability of two machine learning (ML) algorithms based on real-time feature analysis of arterial waveforms obtained from a non-invasive continuous blood pressure system (Finometer^®^) signal to predict the onset of decompensated shock: the compensatory reserve index (CRI) and the compensatory reserve metric (CRM). One hundred ninety-one healthy volunteers underwent progressive simulated hemorrhage using lower body negative pressure (LBNP). The least squares means and standard deviations for each measure were assessed by LBNP level and stratified by tolerance status (high vs. low tolerance to central hypovolemia). Generalized Linear Mixed Models were used to perform repeated measures logistic regression analysis by regressing the onset of decompensated shock on CRI and CRM. Sensitivity and specificity were assessed by calculation of receiver-operating characteristic (ROC) area under the curve (AUC) for CRI and CRM. Values for CRI and CRM were not distinguishable across levels of LBNP independent of LBNP tolerance classification, with CRM ROC AUC (0.9268) being statistically similar (*p* = 0.134) to CRI ROC AUC (0.9164). Both CRI and CRM ML algorithms displayed discriminative ability to predict decompensated shock to include individual subjects with varying levels of tolerance to central hypovolemia. Arterial waveform feature analysis provides a highly sensitive and specific monitoring approach for the detection of ongoing hemorrhage, particularly for those patients at greatest risk for early onset of decompensated shock and requirement for implementation of life-saving interventions.

## 1. Introduction

Sensor technology can play a critical part in the employment of early medical intervention(s) that are associated with improved clinical outcomes in patients suffering from traumatic injury [1]. As such, early intervention relies on early and accurate diagnosis of patient status that can be generated from specific sensors that provide the necessary physiological signals. Such diagnosis has proven historically challenging because of medical monitors that are limited by inadequate measures of standard vital signs that change very little during the compensatory stage of hemorrhage [2]. To address this limitation, there has been emerging technology based on the application of artificial intelligence (AI) to identify real-time changes in the sum total of all physiological mechanisms involved in the compensation for low blood volume states (i.e., central hypovolemia). This physiological phenomenon has been defined as the compensatory reserve, with its measurement relying on a machine learning (ML) approach that incorporates the interrogation of arterial blood pressure waveform features [1,2,3,4,5,6,7]. Reported measurements of the compensatory reserve have consistently proven to provide greater sensitivity in time (i.e., early) and specificity for identifying individual patient status when compared to traditional standard vital signs in both human experimental [2,6,8,9,10,11,12,13] and clinical [14,15,16,17,18,19,20] settings. In this regard, the measurement of the compensatory reserve has proven to be “the most informative ‘vital sign’ to be captured in emergency medical care settings” because of its ability to provide earlier and individualized status of patients with hypovolemia [2].

Despite its repeated accuracy for tracking reductions in central circulating blood volume [1,2,5,8], we are unaware of any evidence that the physiological basis for measuring the capacity to compensate for low circulating blood volume resides in the assessment of arterial waveform morphology without using a specific ML algorithm. We therefore felt it necessary to conduct a systematic comparison of compensatory reserve measurements generated by two entirely different algorithmic approaches. Within this context, we hypothesized that both algorithms designed to evaluate arterial waveform features would yield similar performance for assessing circulating blood volume status based on the evaluation of sensitivity and specificity for tracking the compensatory status of human subjects. Stated more simply, the accuracy of a compensatory reserve measurement should be independent of any ML monitoring approach designed to interrogate waveform features. To test this hypothesis, we compared for the first time two independently generated algorithms developed for measurement of the compensatory reserve based on the application of entirely different ML techniques. We used a common data set of arterial waveforms obtained from a large cohort of human subjects who underwent progressive reductions in central blood volume similar to that experienced during hemorrhage.

## 2. Methods

### 2.1. Subject Volunteers

One hundred nighty-one women (N = 88) and men (N = 103) with a mean (±SD) age of 27 ± 8 years, height of 164 ± 30 cm, and weight of 74.5 ± 16.1 kg volunteered to participate in this investigation after all procedures and potential risks were explained and their written informed consent was obtained. To ensure a state of health, each subject completed a medical history survey and underwent a physical examination before experimentation. For at least 24 h prior to an experiment, participants were instructed to abstain from the use of alcohol, nicotine, caffeine, medications, and/or any drugs that could affect autonomic functions. Female subjects were excluded if they displayed a positive urine pregnancy test during their physical examination. All experimental procedures and protocols were explained to each subject prior to obtaining written informed consent.

### 2.2. Experimental Protocol

A two-group, repeated measures study design was used to compare the ability of the CRI and CRM to predict the onset of decompensated shock. The experimental protocol was designed to determine the tolerance to central hypovolemia of each volunteer subject by applying progressively increasing levels of lower body negative pressure (LBNP). Central hypovolemia induced by LBNP in humans has been shown to result in the integrated activation of compensatory mechanisms comparable to those observed during actual hemorrhage [1,21]. Previous experiments have demonstrated that −30, −60, and −90 mmHg LBNP approximates average blood losses of 450, 1000 and 1600 mL in a 70 kg human [22]. 

Prior to testing, participants were measured with a 3-lead electrocardiogram and non-invasive blood pressure monitor (Finometer^®^ Blood Pressure Monitor, TNO-TPD Biomedical Instrumentation, Amsterdam, The Netherlands) on the left middle finger. During testing, the left hand rested at heart level to ensure accurate representation of central blood pressure, and arterial pressure waveforms were calibrated with a standard manual brachial blood pressure cuff. Hemodynamic data from the non-invasive blood pressure monitor were sampled at 500 Hz and recorded directly with data-acquisition software (WINDAQ, Dataq Instruments, Akron, OH, USA). Recordings from the same arterial waveforms were used to ensure that the calculation of Compensatory Reserve Metric (CRM) and Compensatory Reserve Index (CRI) values were comparable (described in detail below). All subjects were supine for the duration of each testing session and instructed to lie perfectly still to assure the presence of “clean” signals. Participants were placed into the airtight LBNP chamber up to their waist. A seal around the edge of the LBNP chamber was accomplished using a tight-fitting neoprene material around the waist that was also attached to the chamber to allow for the generation of the negative pressure. Negative pressure was generated using a standard vacuum hose, attached to the LBNP chamber that was integrated with a variable autotransformer to incrementally increase suction during the LBNP protocol. Following a baseline resting period of 5 min, each subject underwent exposure to an experimental profile that consisted of progressive LBNP levels at −15, −30, −45, −60, −70, −80, −90, and −100 mmHg for 5 min each. The LBNP protocol was immediately terminated at the time that a subject experienced the onset of clinical decompensated shock as defined by a fall in systolic blood pressure (SBP) to <80 mmHg with concurrent expression of any combination of pre-syncopal symptoms such as nausea, cold sweat, dizziness, or tunnel vision. Release of LBNP to ambient pressure resulted in rapid restoration of central blood volume to the central circulation with concurrent stabilization of hemodynamic stability.

### 2.3. Measuring the Compensatory Reserve

The compensatory reserve is a physiological phenomenon that represents the sum total of all mechanisms that protect against inadequate systemic delivery of oxygen (DO_2_) to the tissues of the body. As such, the compensatory reserve was calculated as the difference in the capacity to compensate for hypovolemia in a resting baseline state (estimated as 100% reserve) and at the onset of hemodynamic instability when the capacity to compensate had been exhausted (i.e., 0% reserve) [1,5,6,7,23,24,25,26]. In this regard, each individual has a finite “reserve” to compensate for low blood volume and flow states. For this investigation, we applied two independently generated ML algorithms for the calculation of an estimated valued of the compensatory reserve: (1) the compensatory reserve index (CRI; Flashback Technologies Inc., Denver, CO, USA) and (2) the compensatory reserve metric (CRM; Mayo Clinic Special Purpose Processor Development Group, Rochester, MN, USA). These algorithms provided state-of-the-art feature-extraction and ML methods for retrospective calculation of compensatory reserve based on changes in morphology of analog arterial pressure waveforms obtained non-invasively with an infrared finger blood pressure signal using the volume clamp technique [1,4,5,6,7,9,10,27].

#### 2.3.1. Compensatory Reserve Index (CRI)

The proprietary CRI algorithm was developed from the application of feature extraction and ML methods used for robotic situational awareness that was translated to vital sign waveform data generated from LBNP experiments [4,7]. The resulting training data included >650,000 training sample waveforms. This approach led to the identification of hundreds of features within each non-invasive arterial waveform that showed a trend of the compensatory phase of central blood volume loss. In this regard, the algorithm was constructed with use of the following generalized equation to calculate an estimate of CRI:

$$\mathrm{CRI}=1-\frac{\mathrm{BLV}}{\mathrm{BLV}@\mathrm{HD}}$$

where BLV is the current blood loss volume of the subject and BLV@HD is the BLV at which the onset of hemodynamic decompensation occurs in that subject. Within this construct, the calculated estimate of CRI relied on an assumption that an individual’s BLV at any given time is known, as well as the individual’s BLV@HD due to reduced central blood volume. The accuracy of this assumption is supported by experiments using non-human primates and human subjects that demonstrated how LBNP closely mimics physiologic responses observed when compared to hemorrhage [1,28,29,30,31]. These direct comparisons allowed for translation of −30, −60, and −90 mmHg LBNP to average equivalents of approximately 450, 1000, and 1600 mL blood loss in a 70 kg human [22,30]. As such, the relationship between LBNP and BLV allowed for an ethically and scientifically justified substitute for modeling the reduction in central blood volume to hemodynamic decompensation in humans using the following calculation to estimate CRI:

$$\mathrm{CRI}=1-\frac{\mathrm{BLV}\left(t\right)}{\mathrm{BLV}@\mathrm{HD}}\approx 1-\frac{\mathrm{LBNP}\left(t\right)}{\mathrm{LBNP}@\mathrm{HD}}$$

where LBNP(t) is the LBNP level that the individual is experiencing at time t and LBNP@HD is the LBNP level at which there is an onset of hemodynamic decompensation in that individual [7].

#### 2.3.2. Compensatory Reserve Metric (CRM)

A detailed description of the CRM algorithm has been previously reported [32]. In summary, a deep one-dimensional (1-D) Convolutional Neural Network (CNN) was trained on arterial waveform data generated from a larger set of LBNP study subjects that included the 191 subjects used for comparison with the CRI. Similar to the approach used to construct the CRI algorithm, the CRM training target was modeled as a series of steps corresponding to the applied LBNP, with an LBNP level used as a target of reduced central circulating blood volume. The final architecture for the CNN was determined using a hyper-optimization search that resulted in the selection of an architecture consisting of an initial input convolutional layer, seven convolutional/pooling layers, two fully connected layers, and a final linear layer. 

**Figure 1:**
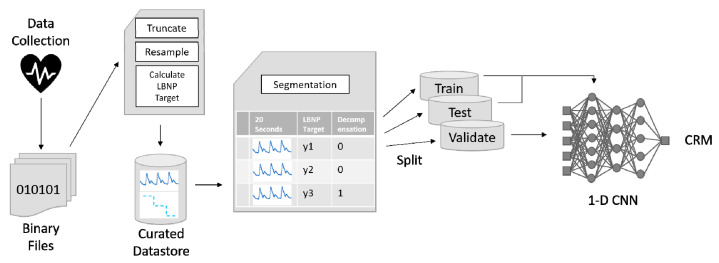
Data-processing pipeline for CRM algorithm development.

The data processing pipeline is shown in Figure 1. The predicted time for onset of hemodynamic decompensation (i.e., decompensated shock) was derived from the release of LBNP. Once the endpoint targets were defined, the analog arterial waveforms recorded from the Finometer^®^ monitor were truncated to the experiment length, beginning after all sensors and equipment were in place and activated for data collection. The end-point of data collection occurred at the time that the subject experienced hemodynamic decompensation (i.e., at the time that the LBNP was released to normal level). Because the subjects were instructed to remain still during the entire data collection process, very few anomalies in the waveforms were revealed, but they were not filtered or altered because it was unclear whether these were introduced by the monitoring equipment or due to an actual physiological cause. The 500 samples per second monitoring frequency of the Finometer^®^ device was reduced to 100 samples per second using 1-dimensional linear interpolation, as it was previously determined that higher frequencies did not produce significantly more accurate results. The waveform for each subject was then normalized to a 0–1 range using min/max scaling and divided into equal segment lengths of 20 s. Each segment could then be used as a separate sample for model training, thus producing a large number of samples from a limited number of original subjects. Each waveform segment was associated with a stepwise CRM training target and a binary flag marking the time of decompensation.

### 2.4. High versus Low Tolerance Classification

Participants were categorized as having high tolerance (HT) or low tolerance (LT) to reductions in circulating central blood volume (i.e., central hypovolemia) using statistical analysis of Kaplan–Meier “survival” curves [21]. By definition, LT participants experienced the onset of decompensated shock prior to completing a LBNP level of 60 mmHg (total protocol time < 1500 s including baseline rest), while HT participants tolerated LBNP levels that exceeded 60 mmHg of LBNP (>1500 s of the total protocol time). However, it should be noted that a single CRI and CRM model was constructed with Finometer^®^ waveform data and applied to all 191 subjects independent of their tolerance to reduced central blood volume (i.e., there are no independent algorithms for HT and LT subjects).

### 2.5. Statistical Analysis

Differences in demographics between HT and LT groups were analyzed using a Student’s *t*-test statistic for independent groups. CRI and CRM LS means with ± standard errors (±SE) were calculated for all 191 subjects (ALL) as well as HT and LT subjects across each of the LBNP levels (baseline to 100% tolerance). To test our hypothesis that the measure of compensatory reserve to predict the onset of decompensated shock would be similar when comparing the CRI and CRM values, generalized estimating equations (GEE) with logit link functions and random effects for subjects were used to perform repeated measures logistic regression analysis. There were two GEE models utilized in this study that regressed the onset of decompensated shock for CRI and CRM. The predicted probabilities outputted from each model were used for comparison in a Receiver Operating Characteristic (ROC) Area Under the Curve (AUC) statistical analysis. The predicted probabilities of onset of decompensated shock for both CRI and CRM were reported by LBNP levels (baseline to 100% tolerance). The probability that any differences between CRI and CRM values were not attributable to chance was analyzed using GEE models with compound symmetry covariance structures and expressed as *p* values. Statistical comparisons were also performed across all levels of LBNP and between LT and HT individuals. In an effort to assess the ability of each algorithm to reach the target LBNP level, amalgamated correlation coefficients (R^2^) were generated from subject group averages of the final CRI and CRM values calculated at the end of each 5 min level during progressive LBNP. In order to compare the strength of the relationships between scaled values of CRI vs. CRM and LBNP, corresponding Pearson correlation coefficients were calculated and converted to z-scores using Fisher’s r to z transformation, and Steiger’s Z tests for dependent samples per each subject class (ALL, HT, and LT).

## 3. Results

For a head-to-head comparison of CRI and CRM, we identified a subset of LBNP study subjects common to validation experiments conducted on CRI [7] and CRM [32]. CRI and CRM were computed every 10 s from the beginning of each experiment until the point of decompensation. Of the 191 subjects used for head-to-head comparisons of the CRI and CRM algorithms, 60 were classified as having low tolerance to central hypovolemia with a mean (±SD) tolerance time of 1286 ± 193 s, while the remaining 131 subjects were classified as having high tolerance with an average tolerance time of 1838 ± 262 s (*p* < 0.001). Demographically, the HT group had a mean (±SD) age (27 ± 8 years), height (163 ± 32 cm), and weight (75.4 ± 15.2 kg) that were statistically indistinguishable (0.617 ≥ *p* ≥ 0.258) from the LT group (27 ± 8 years, 166 ± 27 cm, 72.5 ± 18.0 kg, respectively).

**Figure 2:**
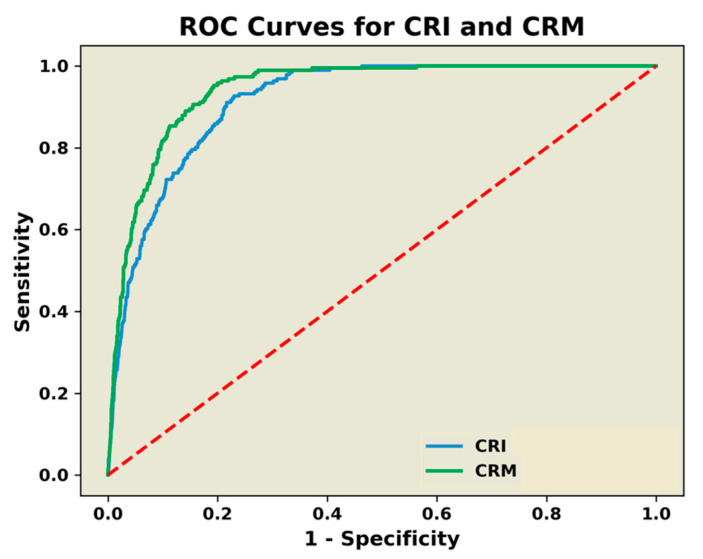
ROC AUC comparisons for prediction of the onset of decompensated shock between the compensatory reserve index (CRI—blue line) algorithm and the compensatory reserve metric (CRM—green line) algorithm. Diagonal broken red line represents a random guess threshold (i.e., no-discrimination line at 0.5).

As presented in Figure 2, the GEE analysis produced an ROC AUC (±MSE) for predicting the onset of hemodynamic decompensation (decompensated shock) of 0.9164 (0.0066, 95% CI = 0.903–0.929) for CRI compared to the CRM ROC AUC of 0.9268 (0.0059, 95% CI = 0.915–0.938). The CRM ROC AUC was statistically greater at *p* = 0.104.

**Figure 3:**
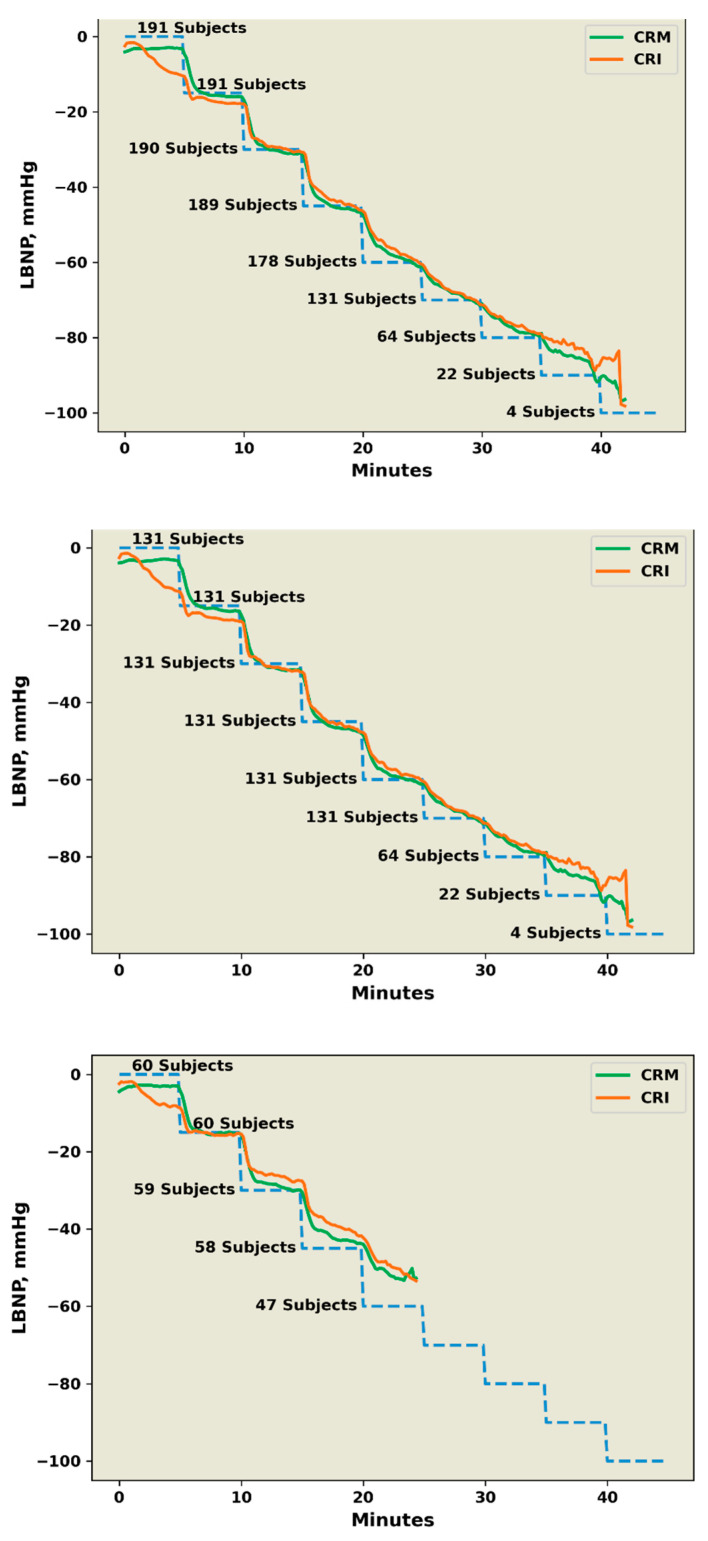
Average responses of compensatory reserve estimated by the CRI (orange line) and CRM (green line) algorithms for all 191 subjects (**upper** panel), 131 high-tolerance subjects (**middle** panel B), and 60 low-tolerance subjects (**lower** panel). The LBNP profile steps used for model development as a target of reduced central circulating blood volume are indicated by the blue broken line (labeled on the *y*-axis).

Comparisons of average responses of compensatory reserve estimated by the CRI and CRM algorithms during progressive stepwise reductions in central blood volume in all 191 subjects are presented in Figure 3 (upper panel). The number of subjects who progressed through each stage of LBNP is also presented. A model comparing CRI with CRM irrespective of time showed the calculated responses generated from two algorithms to be statistically similar (*p* = 0.114). However, a statistical comparison of LBNP-averaged values between CRI and CRM as functions of time revealed that the interaction between CRI and CRM with time was statistically different; a finding consistent with average baseline rest values of 96 ± 6% for CRM compared to 86 ± 6% for CRI (*p* < 0.001). Statistical analysis of estimated compensatory reserve responses for HT (Figure 3, middle panel) and LT (Figure 3, bottom panel) groups revealed statistically similar results (*p* = 0.626) compared to those generated by analysis of the entire group of subjects.

**Figure 4:**
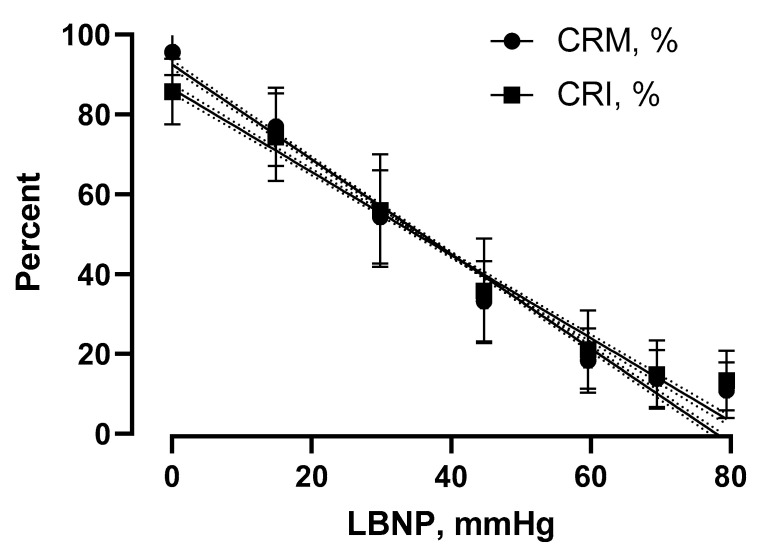
Plots of linear regressions calculated between progressive LBNP levels and measurements of compensatory reserve generated from CRM (circles) and CRI (squares) algorithms. Values are the mean ± SD calculated at the end of each 5 min step of LBNP from all 191 data sets presented in Figure 3 (upper panel).

The amalgamated correlation coefficients (R^2^) between values of CRI and CRM across the progression of LBNP are presented in Table 1 and Figure 4. The strength of the relationships between LBNP and estimated compensatory reserve values were not statistically different between CRM and CRI for all 191 subjects (*p* = 0.344) and for HT subjects (*p* = 0.232). However, the strength of the relationship between LBNP and compensatory reserve values for LT subjects was statistically different (*p* < 0.0001) between CRM and CRI, with CRM displaying a stronger negative R^2^ than that for CRI.

## 4. Discussion

Data generated from numerous experimental and clinical investigations have demonstrated that measurement of the compensatory reserve based on real-time assessment of changes in arterial waveform features provides an earlier and more specific metric of patient status during conditions of reduced central blood volume compared to standard methods of medical monitoring. Within this conceptual framework, we hypothesized that algorithmic approaches that apply ML techniques to the same training library of analog signals in a model of progressive central hypovolemia should generate similar compensatory reserve values and predictive capabilities for the onset of hemodynamic decompensation. The results of this investigation support our hypothesis by demonstrating statistically similar sensitivity, specificity, and values of compensatory reserve across a wide range of LBNP levels independent of differing ML approaches used in constructing the CRI and CRM algorithms. The significance of this finding is that it reflects the basic fundamental premise that the morphology of the arterial waveform represents the most accurate approach to measuring the integration of all compensatory mechanisms that best predict the onset of circulatory shock in an individual patient [1,2,3,5]. In other words, it is critical to appreciate that an efficacious algorithm for monitoring the capacity to compensate for reduced circulating blood volume is most dependent on physiological signals that best represent a specific clinical condition rather than the algorithmic approach per se.

A ROC AUC ≥ 0.9 can be considered as highly accurate compared to moderately accurate values of 0.7 < AUC ≤ 0.9 [33]. In this regard, the results of the present investigation are consistent with ROC AUC values ≥ 0.9 previously reported for predicting the presence of ongoing hemorrhage or the onset of hemodynamic decompensation using a measurement of CRI or CRM compared to moderate accuracy provided by standard vital sign measurements [2,9,10,11,12,13,18,34]. These observations should be expected given that the added value of arterial feature waveform analysis to the assessment of clinical status in hypovolemic patients has been well documented [1,2,35,36,37,38]. As such, a high accuracy associated with the measurement of the compensatory reserve underscores the importance of including ML technologies for obtaining real-time changes in arterial waveform morphology for sensor development designed to advance medical monitoring capabilities.

The average group response measured with the LBNP-scaled CRM algorithm across levels of decreasing central blood volume (i.e., LBNP) was statistically different from LBNP-scaled CRI responses. With nearly identical patterns across the LBNP profile, the finding of a statistical difference is most likely attributed to differences in patterns and average values of compensatory reserve measured with CRI and CRM algorithms during the first stage of the protocol (Figure 2, all panels). While CRM was stable at baseline rest (initial 5 min protocol level), the CRI algorithm displayed instability with a progressive reduction in the absence of change in central volume. This comparison may necessitate additional investigation into the stability of the FDA-cleared CRI algorithm.

High sensitivity, specificity, and accuracy of a real-time measurement of the compensatory reserve to predict the onset of decompensated shock was determined to be statistically similar for the entire subject population based on ROC AUC analysis. The algorithms also demonstrated a “steady state” pattern in compensatory reserve during each of the early stages of reduced central blood volume (Figure 2). However, the inability to maintain a steady-state compensatory reserve after the −45 mmHg LBNP level (i.e., third step) translates to a compromised capacity to sustain adequate tissue oxygen delivery after an average loss of approximately 700 to 750 mL of circulating blood volume for a 75 kg individual [1,22]. However, the exceptionally high correlation coefficients generated from population averages of CRI and CRM at the end of each LBNP level provide the first compelling evidence to support the notion that 5 min is an adequate time for the algorithms to provide an accurate and validated measurement of the compensatory status of an individual.

A unique characteristic of the data sets used in the development of both CRI and CRM algorithms was the ability to include the classification of individuals with varying tolerances to reductions in central blood volume. As such, this large database consisting of analog arterial waveforms provided for the first time an ability to construct ML algorithms that are designed to distinguish “good” from “poor” compensators. This unique capability was further supported by the high correlation coefficients generated between CRI and CRM values with LBNP in this study. In this regard, both CRI and CRM accurately tracked the reduction in compensatory reserve in individuals independent of their capacity to compensate for progressive central hypovolemia induced by increasing levels of LBNP. This observation supports the conservation of the physiological compensatory response across individuals regardless of the magnitude of an individual’s absolute compensatory reserve. Although an average low-tolerant individual reaches decompensation approximately twice as quickly as a high-tolerant individual [21], CRM and CRI were able to accurately correlate compensation capacity to simulated blood volume reduction for both groups of individuals. Indeed, being able to distinguish patients with relatively low tolerance to reduced central blood volume is key to providing early diagnosis of and intervention for those individuals at highest risk for the onset of decompensated shock, characteristics that are critical to the development of efficacious wearable sensors. In this regard, the CRM appears to provide the most efficacious algorithm based on its statistically stronger relationship between reduced central blood volume and compensatory reserve measures in LT subjects. We are unaware of any other advanced technology applying ML that provides such a capability for translation of precision medicine to patient monitoring. The performance results from both CRI and CRM algorithms with high sensitivity, specificity, and accuracy presented in this paper underscore the importance of accumulating data sets from healthy subjects exposed to an experimental protocol designed to elicit hemodynamic decompensation in all subjects prior to validating the algorithms with an application in patients [14,15,17,18,19,20,34]. Simply put, consistent success in accurately assessing the clinical status of patients with compromised circulating blood volume reflects the importance of generating algorithms that are based on the physiology of healthy individuals.

Although efforts have been made to use the same data sets for algorithm comparisons, there were subtle differences in the approaches to develop the CRI and CRM algorithms that could have potentially influenced the interpretation of results generated from this investigation. For instance, the experimental design allowed for the same subset of 191 subjects to be used for algorithm performance analysis of ROC AUC calculations and compensatory reserve responses during progressive reductions in central blood volume (Figure 1, Figure 2 and Figure 3). However, subject data sets collected during the initial 184 LBNP experiments were used to develop the CRI algorithm [7], compared to a larger common data set with additional subjects used 6 years later to develop the CRM algorithm [32]. Additionally, CRI values were generated from averaging calculations across a 30-beat sliding window [7], while the CRM values were calculated based on 20 s waveform segments [32]. Despite these subtle differences in the specific approach for algorithm development, the remarkable similarity in CRI and CRM performance for calculating individual compensatory reserve values with high sensitivity and specificity reflects the incredible influence and stability imparted by the use of a database consisting of hundreds of thousands of arterial waveforms for “learning”.

*Translation of compensatory reserve algorithms into clinical practice*. A potential limitation to the clinical application of this work is that compensatory reserve algorithms were developed based on data collected from healthy subjects undergoing experimentally controlled progressive central hypovolemia rather than patients with actual hemorrhage. To address this issue, there have been clinical investigations designed to examine the sensitivity and specificity of compensatory reserve algorithms in trauma patients with severe blood loss [12,15,16,17,34,39]. The results of these studies consistently demonstrate a clear superiority of compensatory reserve algorithms when ROC AUC is compared with standard clinical vital signs (Table 2). These clinical results support the fundamental physiological basis of the compensatory reserve algorithms with accurate application across a wide spectrum of health and disease.

## 5. Conclusions

As technology advances with the application of novel monitoring capabilities designed to facilitate early and accurate diagnosis and triage of individual patients, the incorporation of sensors capable of supporting compensatory reserve measurements can ensure that patients who require emergency medical care prioritization receive timely and appropriate treatment interventions. As such, the development and availability of a single advanced monitoring system that includes wearable sensors capable of capturing analog arterial waveforms or photoplethysmographic signals, and integrates them with the application of ML algorithms, will prove essential to advancing decision support with the goal of optimizing health, safety, and wellbeing in prehospital and emergency room settings. In this regard, it is important to recognize that wearable sensors must be designed with the capability to capture arterial waveform analog signals in order to provide the clinical caregiver with real-time assessment of patient status, including the highest sensitivity, specificity, and accuracy for making clinical decisions easier within time-critical challenging situations. Finally, further research efforts should reveal that such sensor systems and associated algorithms such as the CRI and CRM may be applied to the diagnosis and/or management of other cardiovascular conditions that might compromise the health, wellbeing, or safety of an individual (e.g., dehydration, hypoxia, sepsis, heart failure, pneumothorax, and physical fatigue).

## Figures and Tables

**Table 1:** Amalgamated correlation coefficients (R^2^) between LBNP and CRM and CRI for all subjects and those classified as having high (HT) and low (LT) tolerance to central hypovolemia.

		CRM, %	CRI, %	*p* Value
	N	R^2^	R^2^	
All subjects	191	0.958	0.978	0.344
HT subjects	131	0.965	0.980	0.232
LT subjects	60	0.999	0.991	<0.0001

**Table 2:** Receiver-Operating Characteristic (ROC) Area Under the Curve (AUC) values for CRM compared to various standard vital signs used for assessing the clinical status of bleeding trauma patients.

References	N	Clinical Condition	CRM	SBP	HR	PP	SI	Lac
Nadler et al. [16]	230	Blood Donation	0.84	0.60	0.73	0.51	0.64	-
Stewart et al. [12]	122	Blood Donation	0.90	0.84	0.55	-	-	-
Mackenzie et al. [39]	556	Trauma Hemorrhage	0.78–0.89	-	0.56–0.62	-	-	-
Stewart et al. [34]	44	Trauma Hemorrhage	0.97	0.81	0.64	-	0.74	0.73
Benov et al. [17]	31	GI Bleeding	0.79	0.62	0.60	0.36	-	-
Johnson et al. [15]	89	Trauma Hemorrhage	0.83	0.62	-	-	-	-

CRM, compensatory reserve measurement; SBP, systolic blood pressure; HR, heart rate; PP, pulse pressure; SI, shock index; Lac, blood lactate.

## Data Availability

The data presented in this study are not publicly available because they have been collected and maintained in a government-controlled database that is located at the US Army Institute of Surgical Research. As such, these data can be made available through the development of a Corroborative Research & Development Agreement (CRADA) with the corresponding author.
